# Anti-reflux mucosectomy (ARMS) for refractory gastroesophageal reflux disease

**DOI:** 10.1186/s40001-024-01789-5

**Published:** 2024-03-18

**Authors:** Xian Zhu, Jianwei Shen

**Affiliations:** Department of Gastroenterology, Ningbo Medical Center Lihuili Hospital, 1111 Jiangnan Road, Ningbo, 315000 China

**Keywords:** Anti-reflux mucosectomy, Refractory gastroesophageal reflux disease, Endoscopic antireflux therapy

## Abstract

Gastroesophageal reflux disease (GERD) is one of the most common diseases seen by gastroenterologists worldwide. A significant proportion of patients have a suboptimal response to acid inhibitors, especially proton pump inhibitors and potassium-competitive acid blockers. Due to concerns regarding the safety of long-term medication, many patients are unwilling to take lifelong medication. Endoscopic antireflux management offers a minimally invasive option for GERD patients. In recent decades, there have been several endoscopic antireflux therapies, including radiofrequency therapy, transoral fundoplication, and mucosal resection or mucosal ablation. Of these, antireflux mucosectomy (ARMS) is an effective and safe therapy for refractory GERD. This review provides an updated summary of antireflux mucosectomy.

## Introduction

Gastroesophageal reflux disease (GERD) is a common condition in which the reflux of gastric contents into the esophagus can cause uncomfortable symptoms and/or complications [1]. GERD is highly prevalent all over the world, with approximately 20% of the adult population in the western world experiencing it [2]. The varied symptoms of GERD include esophageal and extraesophageal manifestations [1]. The recurrent symptoms make patients anxious. Long-standing GERD is associated with an increased risk of inflammation of the esophagus, Barrett’s esophagus and esophageal cancer [3, 4]. GERD threatens the quality of life and poses an increasing public health burden worldwide [5, 6].

Management of GERD includes diet and lifestyle modifications, medications , surgery and endoscopic therapies. Acid suppressors, especially proton pump inhibitors and potassium-competitive acid blockers, are the backbone of medical therapy for GERD. However, there are still a considerable proportion of patients who are refractory to acid suppressions, who have a contraindication for such medications or who cannot tolerate long-term drugs. There is no consensus on the definition of refractory GERD worldwide, and the term “refractory GERD” is described when symptoms have not responded partially or completely to a standard dose of proton pump inhibitor therapy after a sufficient period of therapy [7]. In addition, chronic PPI use may put people at risk of experiencing drug interactions and increase the risk of the PPI-associated adverse events [PAAEs] [8].

Surgery is one of the treatments for patients with refractory GERD who have failed medical therapy. The objective of antireflux surgery is to anatomically restore the antireflux barrier. Laparoscopic fundoplication is the current standard antireflux surgery. However, both acute and prolonged complications can occur after laparoscopic fundoplication. Approximately 50% of patients have acute-onset dysphagia, 10% of patients suffer from postfundoplication stenosis, 10–32% of patients experience gas-bloating syndrome, and 18–33% of patients have diarrhea [9]. There are still patients who require acid-suppressive medications several years after antireflux surgery. In 2022, the American Gastroenterological Association Institute, in their clinical guidelines for the management of gastroesophageal reflux disease, recommended endoscopic antireflux procedures, including magnetic sphincter augmentation (MSA) and traditional incisionless fundoplication (TIF), as an option for treatment [1]. Anti-reflux mucosectomy (ARMS) is a new endoscopic strategy for refractory GERD first reported by Inoue et al. [10]. In this review, we will introduce ARMS-related endoscopic antireflux techniques and evaluate the efficacy of ARMS, factors affecting the efficacy of ARMS and complications of ARMS.

### The origin and research status of ARMS

Antireflux mucosectomy (ARMS) was first reported by Inoue et al. in 2014 as a new endoscopic treatment for refractory GERD. In 2003, Inoue et al. reported a patient with high-grade dysplasia (HGD) in a short segment of Barrett’s esophagus [10]. Ten years after endoscopic mucosal resection, the patient remained asymptomatic, without requiring PPI therapy. This case suggests that ARMS may be an effective antireflux endoscopic operation. Subsequently, Inoue et al. refined the method and applied it in a case series study published in 2014 that showed that ARMS could be effective in improving the symptoms and DeMeester score of GERD patients [11]. Since the advent of ARMS, there have been several prospective and retrospective studies to evaluate its efficacy.

### ARMS and other endoscopic anti-reflux techniques

**Fig. 1:**
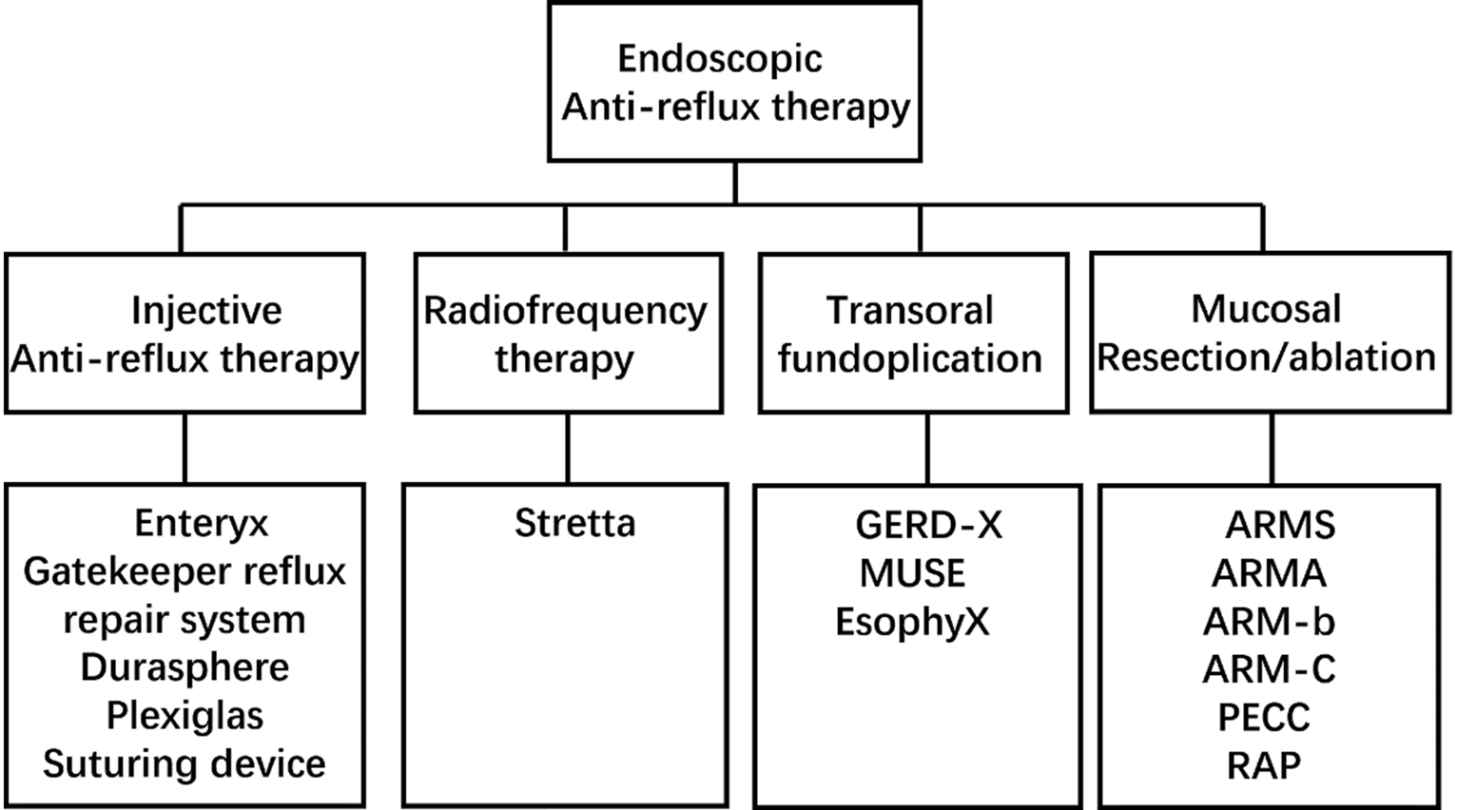
The classifications of endoscopic anti-reflux therapy

With the development of endoscopic technology, an increasing number of GERD endoscopic therapy methods have been proposed. These methods can be broadly divided into four categories (Fig. 1) based on the remodeling of anti-reflux barrier mechanisms [12]. The first method is to inject the injectable agents into the esophagogastric junction to strengthen the anti-reflux barrier (involving Enteryx, the Gatekeeper reflux repair system, Durasphere, Plexiglas and a suturing device). The second method is the application of radiofrequency energy near the lower esophageal sphincter (LES) and the gastric cardia to improve its pressure (Stretta). The third method is endoscopic fundoplication aiming to reconstruct the LES (GERD-X, MUSE, Esophyx). The last is mucosal resection/ablation/constriction of the esophagogastric junction (EGJ) to achieve fundoplication. Due to its safety issues and poor efficacy, the first method is not currently available. For the second and third methods, the clinical application may be limited due to the need for special instruments. According to the ACG Clinical Guideline for the Diagnosis and Management of Gastroesophageal Reflux Disease [1], data on the efficacy of radiofrequency energy (Stretta) as an anti-reflux procedure are inconsistent and highly variable, and it cannot be recommended as an alternative to medical or surgical anti-reflux therapies. The last method seems to be an effective, simple and well-tolerated endoscopic treatment strategy for refractory GERD [13]. The last method includes ARMS using endoscopic submucosal dissection (ESD) or endoscopic mucosal resection (EMR), banded anti-reflux mucosectomy (ARM‑b), anti-reflux mucosal ablation (ARMA), and peroral endoscopic cardial constriction (PECC).

### Different methods of ARMS

**Fig. 2:**
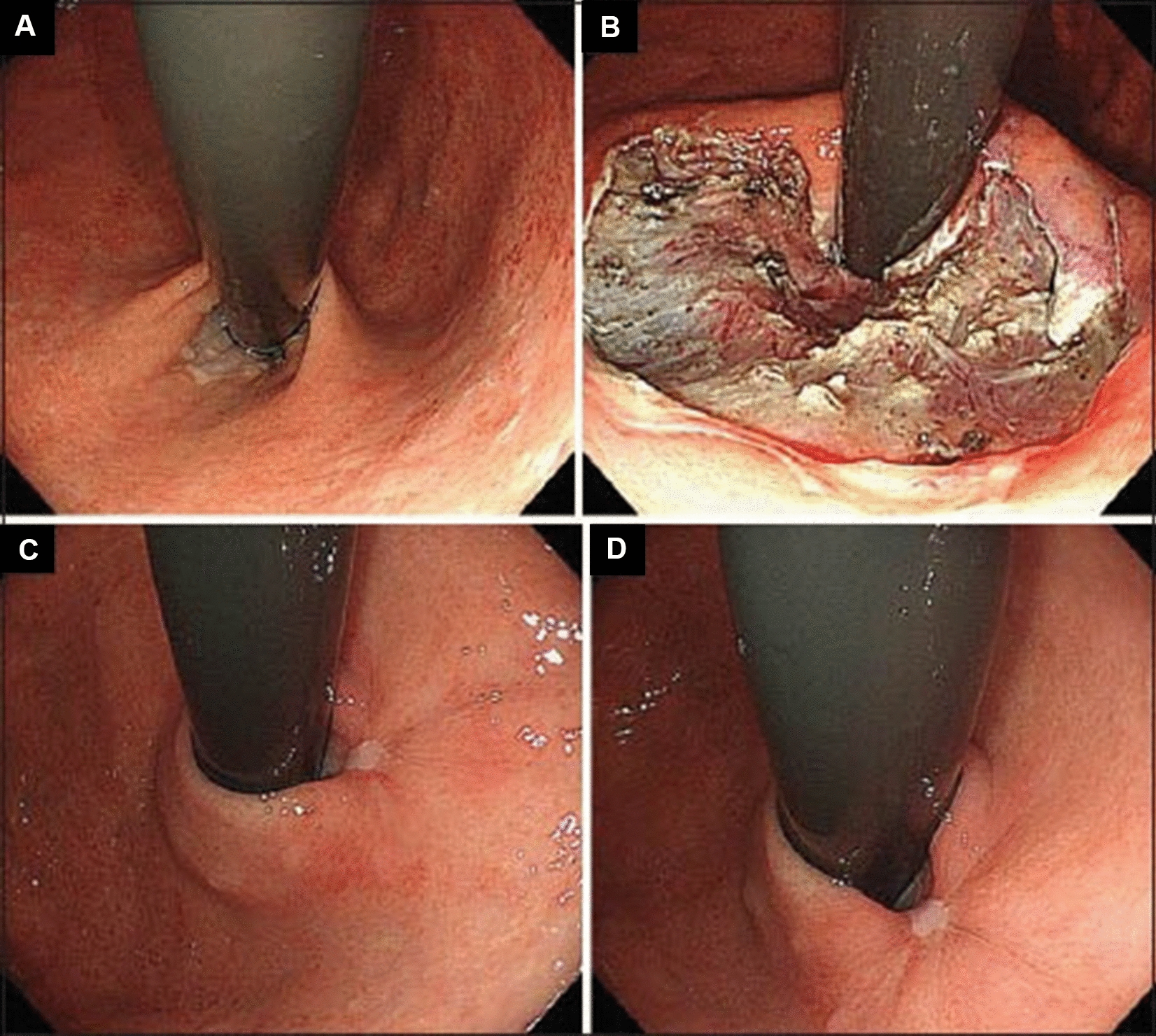
Endoscopic anti-reflux mucosectomy (ARMS). **A **Before ARMS. **B** Immediately after procedure. There is a two-thirds circumferential artificial ulcer at lesser curve after ARMS, and the mucosal flap valve at greater curve was preserved. **C**, **D** Appearance at 2 months. Mucosal valve was well-defined at the lesser curve

**Fig. 3:**
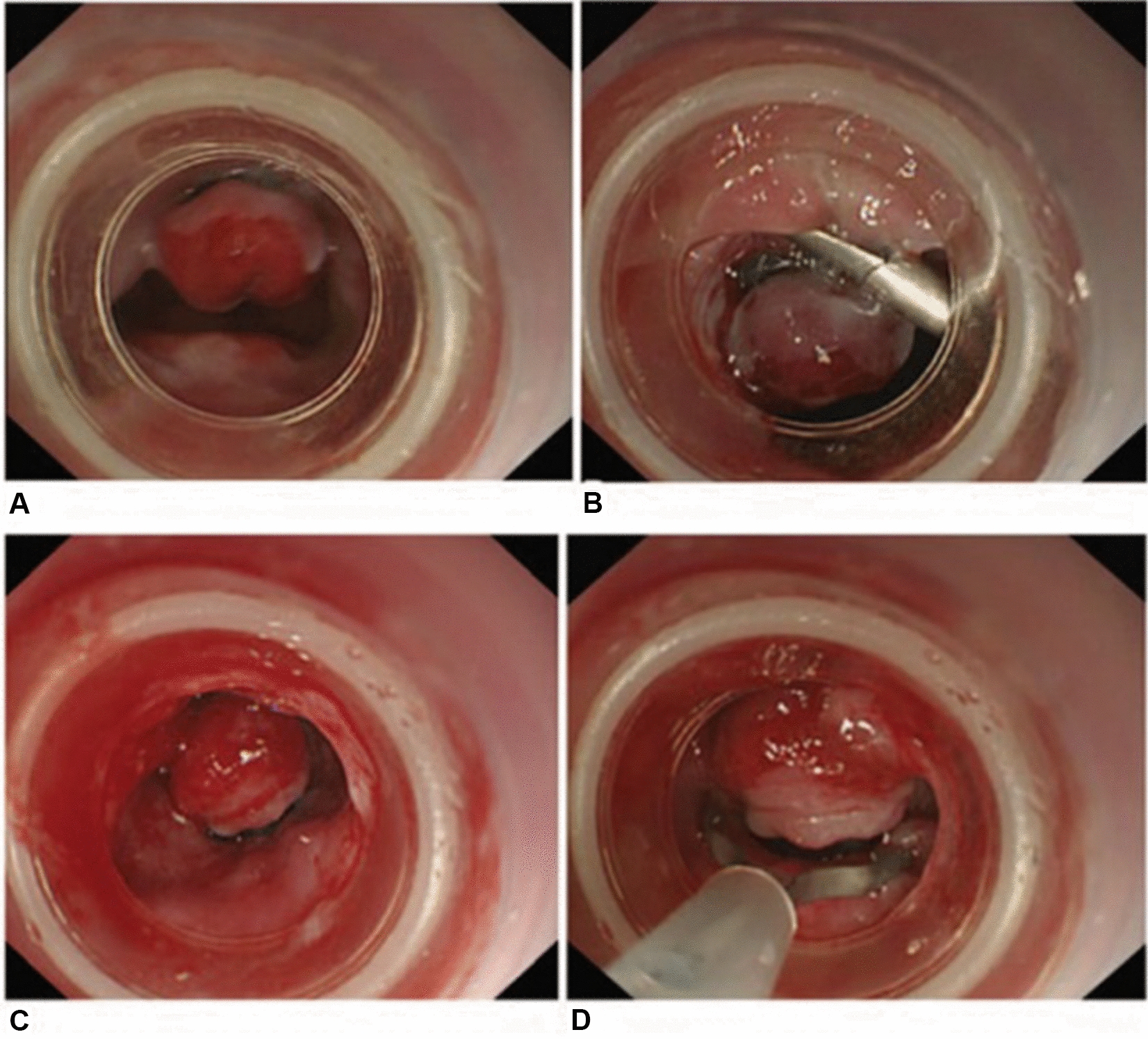
Peroral endoscopic cardial constriction (PECC). **A**,** C** Place two single-band ligation devices at the greater curve and lesser curve of cardia. **B**, **D** Fix two resolution clips at the ends of the ligation devices

**Fig. 4:**
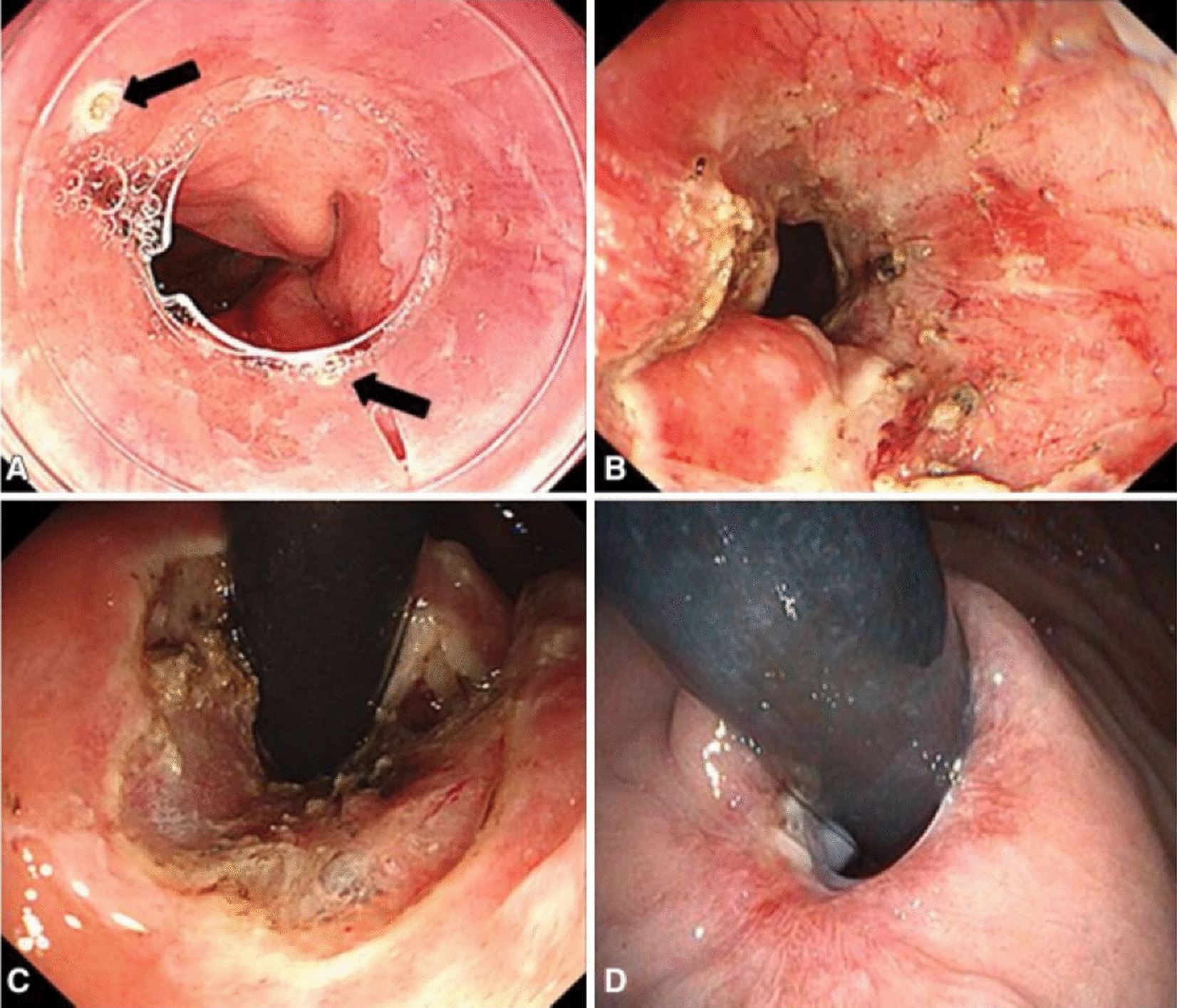
Anti-reflux mucosectomy using cap (ARMS-C). **A** Before ARMS-C. Arrows show the lesion marked with argon plasma coagulation at the 10 o’clock and 6 o’clock. **B**, **C** After the EMR-C method, the mucosa was resected at approximately 270°. **D** Six months after ARMS-C

**Fig. 5:**
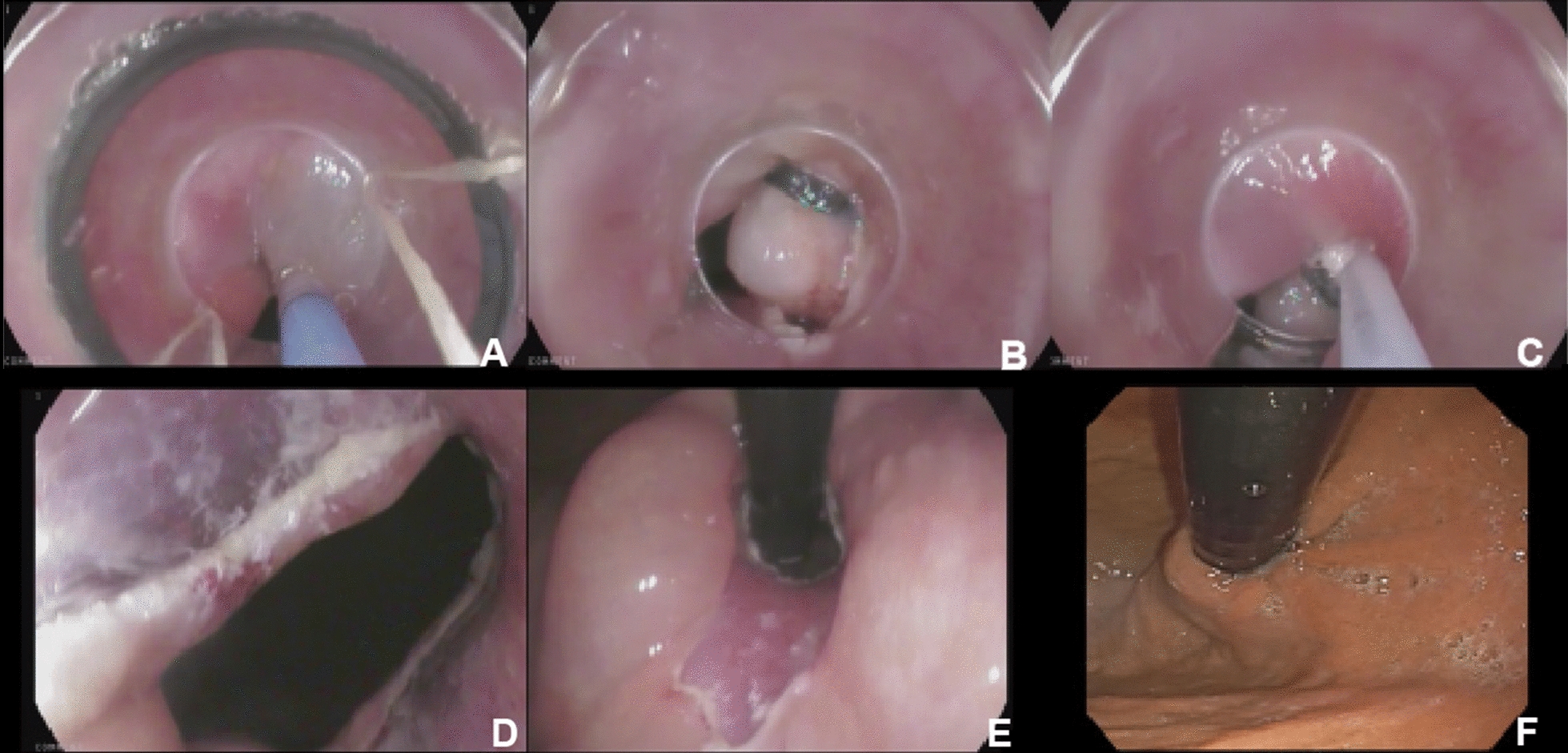
Endoscopic antireflux mucosectomy band (ARM-b) technique. **a** Submucosal injection. **b** Band ligation. **c** Mucoesctomy under the rubber. **d** Front view of the mucosectomy of the cardia. **e** Retroflexion view of the mucosectomy of the cardia. **f** Appearance at 3 months

**Fig. 6:**
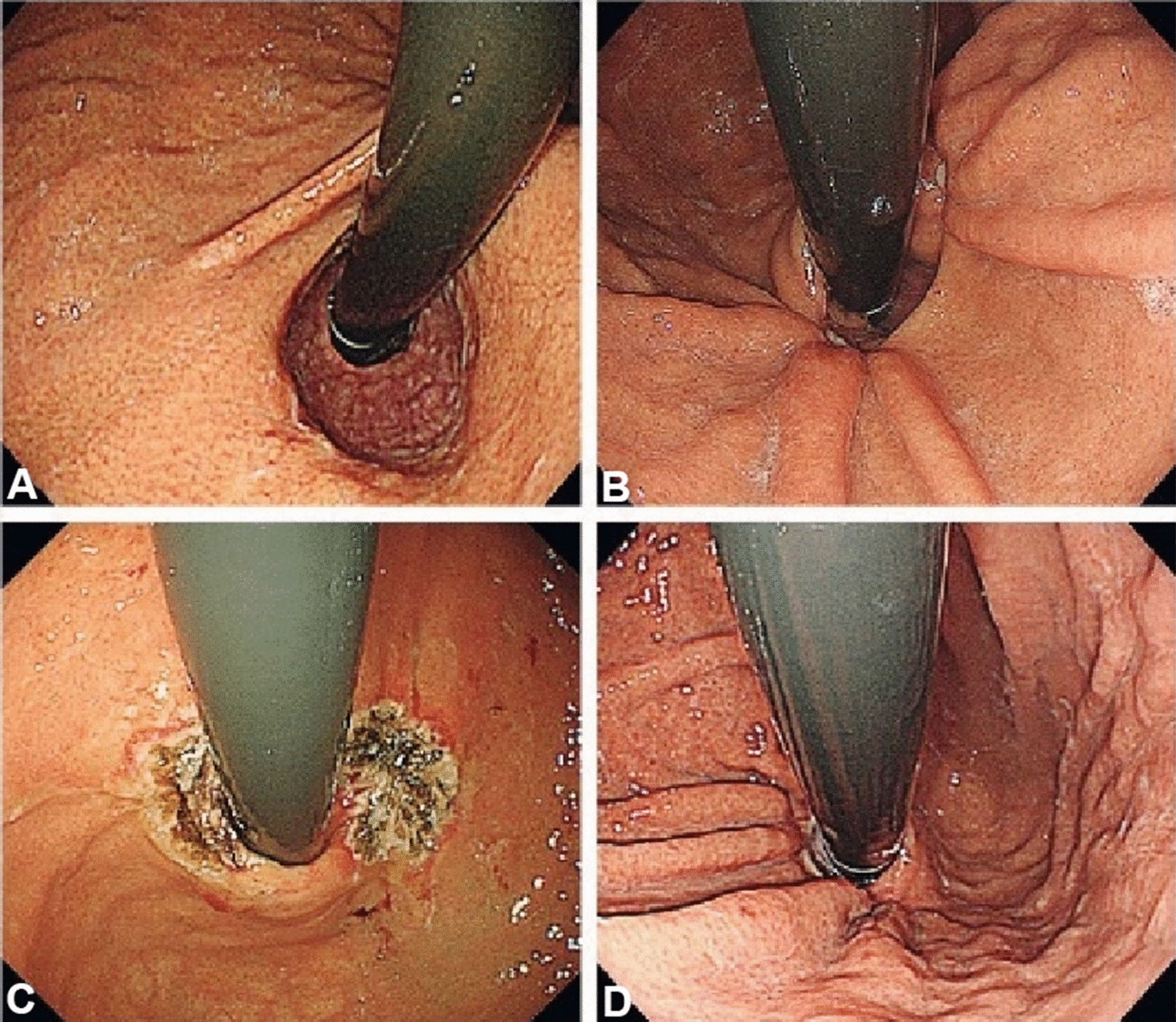
Anti-reflux mucosal ablation (ARMA) treatment. **a** Before procedure. **b** Post-ARMS.**c** Immediately after procedure. **d** Appearance at 1-month post-ARMA. Mucosal flap valve was re-shaped

The aim of anti-reflux mucosectomy (ARMS) is to rebuild the mucosal flap valve by submucosal fibrosis after mucosectomy at the esophagogastric junction (EGJ). According to past studies, the specific operations are different. Inoue et al. [11] reported their pilot study in 2014; 2 patients underwent total circumferential resection, and the subsequent 8 patients underwent crescentic ARMS conducted with the standardized techniques of endoscopic mucosal resection (EMR)/endoscopic submucosal dissection (ESD) (Fig. 2 [11]). Benias et al. [14] evaluated resection and plication (RAP) antireflux, which comprised semicircumferential mucosectomy along with full-thickness plication of the lower esophageal sphincter (LES) and cardia. Hu et al. [15] first reported peroral endoscopic cardial constriction (PECC) (Fig. 3 [15]) in gastroesophageal reflux disease. PECC is simple and easy to apply with a shorter operation time. However, the main factor influencing the efficacy was the depth of ligation. Patil et al. [16] reported ARMS using cap-assisted endoscopic mucosal resection (AMRS-C) (Fig. 4 [17]). Hedberg et al. [18] first reported the use of an antireflux mucosectomy band (ARM-b) (Fig. 5 [19]) in the treatment of refractory gastroesophageal reflux disease. Inoue et al. [20] reported antireflux mucosal ablation (ARMA) (Fig. 6 [20]). Mucosal ablation was performed using the triangle-tip knife J in spray coagulation mode after markings were placed around the cardia and a submucosal cushion was injected. The strength of ARMA is that it can be repeated regardless of the presence of fibrosis from previous therapies. ARMA can be performed in patients who have failed ARMS or who are hesitant to undergo laparoscopic antireflux surgery (LARS). In addition, ARMA does not require specific expensive devices. ARMA improves the flap valve grade and ultimately resolves the patient’s symptoms. The success of ARMS is likely related to its ability to cause submucosal fibrosis at the LES. ARMS prevents the frequent occurrence of transient lower esophageal relaxation (TLESR). Therefore, ARMS techniques are more widely used in anti-reflux endoscopic therapy for refractory gastroesophageal reflux disease.

### How to evaluate the efficacy of ARMS

#### Evaluation of clinical symptoms

Symptom improvement is one of the important criteria for assessing effectiveness in most related studies. It has been reported that reflux symptoms significantly improve after endoscopic treatment (ARMS) in many studies [13]. There are many symptom-related questionnaires, including GERD health-related quality of life (GERD-HRQL), GERD-questionnaire (GERD-Q), frequency scale for symptoms of GERD (FSSG), SF-12 score [19], and reflux severity index (RSI) [18]. There are also a few studies making use of the Los Angeles classification of esophagitis to evaluate the tools used to grade esophagitis [21]. Of these, the GERD-HRQL is the most frequently used questionnaire. Nine of all the studies in Table 1 have made use of GERD-HRQL. Due to different symptom assessments, it is difficult to compare the results of different studies. In addition, symptom questionnaires are subjective indicators that are greatly affected by patients. According to the results of the symptom-related questionnaires, almost all studies showed improvement in scores after AMRS .

**Table 1:** Study and population characteristics

Author, year	Technique	No. patients	Age, mean years (SD)	Mean follow-up time	Clinical success		Partial clinical success	Complete clinical success	24 h esophageal pH monitoring					
									DeMeester score mean (SD)		Mean esophageal acid exposure time (%) (Ph＜4) (SD)			
									Preoperation	Postoperation	Preoperation			Postoperation
Inoue et al. [12], 2014	ARMS	10	NR	2 months	10		0	10	NR		29.1			3.1
Ota et al. [22], 2014	ARMS	13	NR	6 months	12	0		12	NR		10.4 (±15.5)			7.4 (±10.6)
Bapaye et al. [25], 2017	ARMS	15	40.8 (19.2)	1 months	15		4	11	85.8	5.9	NR			
Benias et al. [15], 2018	RAP	10	56.5	5–27 months	10		0	10	NR		NR			
Hu et al. [16], 2018	PECC	16	53	3, 6 months	16		0	16	125.5 (±89.64)	20.32 (±15.22)	35.55 (±26.2)			4.72% (±3.78%)
Patil et al. [17], 2019	ARMS-C (cap-EMR)	62	36 (9.9)	2, 6, 12 months	55		12	43	76.8 (18.3)	14.3 (6.1)	NR			
Hedberg et al. [19], 2019	ARM-b	19	57.1	3 week, 6 months	13		0	13	NR		NR			
Prasad et al. [32], 2019	ARMS	11	41–60	6 months	8		0	8	NR		NR			
Yoo et al. [18], 2020	ARMS-C	33	51.3 (16.3)	6 months	31		10	21	14.3 (10.9)	7.7 (9,4)	3.1(3.1)		1.8 (2.4)	
Monino et al. [27], 2020	ARM-b	21	56.78 (14.47)	5, 10 months	16		4	12	NR		NR			
Inoue et al. [21], 2020	ARMA	12	54.4	2 months	12	0		12	33.5	2.8	9	0.5		
Hernandez et al. [24], 2020	ARAT(ARMA)	108	36.5	3,6,12,24,36 months	96	0		96	42.5	9.1	18.8	2.8		
Debourdeau et al. [20], 2020	ARMS-b	6	44(7.5)	3 months	3	2		1	NR		NR			
Wong et al. [28], 2020	ARMS-b	33	55 (17)	6 months, 1 year, 2 years	30	0		30	NR		NR			
Sumi et al. [23], 2021	ARMS	109	54 (15.7)	2–6 months, 1 year	42		0	42	64.4 (75.7)	24.9 (36)	20.8 (24.3)	6.9 (10.4)		

Author, year	GERD-HRQL score		GERD-Q score mean (SD)		Adverse events			The use of acid inhibitors	Hill grade					
					Dysphagia	Bleeding	Others							
	Preoperation	Preoperation	Preoperation	Preoperation					Post-operation					
Inoue et al. [12], 2014	NR		NR		2	NR	NR	All discontinued	Grade I					
Ota et al. [22], 2014	NR		NR		1	NR	NR	3 discontinued 3 reduced in dose 6 at the usual dose	NR					
Bapaye et al. [25], 2017	40.4	7.6	NR		1	0	2	NR	NR					
Benias et al. [15], 2018	26.6 (±3.9)	4.3 (±2.4)	NR		1	NR	NR	6 discontinued 4 reduced in dose	Grade I					
Hu et al. [16], 2018	36.5	10	NR		3	0	1	NR	NR					
Patil et al. [17], 2019	NR		10.6 (1.9)	3.4 (1.5)	5	0	4	43 discontinued 12 reduced in dose 7 at the usual dose	NR					
Hedberg et al. [19], 2019	Improved		NR		3	1	1	13 discontinued	NR					
Prasad et al. [32], 2019	NR		NR		0	0	0	NR	NR					
Yoo et al. [18], 2020	NR		11.1 (3.1)	6.8 (3.1)	2	0	0	21 discontinued 10 reduced in dose	Grade I					
Monino et al. [27], 2020	25.6 (8.8)	16.8 (6.4)	12.5 (1.5)	9 (2)	3	1	0	3 months 76% Decrease/discontinue 6 months 72% Decreased/discontinued	19 improved					
Inoue et al. [21], 2020	30.5	12	NR		1	0	0	5 discontinued	Grade I (1.9–0.5)					
Hernandez et al. [24], 2020	36.5	10	NR		14	0	0	78.6% 3 years discontinued	Grade I					
Debourdeau et al. [20], 2020	30.6 (7.7)	6.8 (3.7)	13.3 (1.1)	6.2 (4.0)	1	1	0	1 discontinued 2 reduced in dose 3 at the usual dose	NR					
Wong et al. [28], 2020	16 (12)	6 (7.1)	NR		3	1	1	90.9% discontinued	Grade I					
Sumi et al. [23], 2021	NR		NR		14	2	1	50% 1 year discontinued	NR					

*GERD-HRQL*, GERD-Health Related Quality of Life, *GERD-Q* GERD-questionnaire, *mon* month, *SD* standard deviation, *RAP* resection and plication, *PECC* Peroral endoscopic cardinal constriction, *ARMS-C* anti-reflux mucosectomy using cap-assisted endoscopic mucosal resection (EMR-C), *ARAT* antireflux ablation therapy, *ARMA* anti-reflux mucosal ablation, *ARMS-b* antireflux mucosectomy band, *NR* not reported

#### 24-h esophageal pH monitoring

The DeMeester score and esophageal acid exposure time (AET) are the main objective data of the antireflux effect. Compared to subjective scoring systems, the results of the DeMeester score and AET are more reliable. The questionnaire scores are influenced by the placebo effect of undergoing treatment; however, objective measures of AET and DeMeester scores do not have this limitation. As shown in Table 1, the DeMeester score was used in seven studies to assess the efficacy of ARMS. The DeMeester scores were significantly improved after the ARMS in all seven studies [11, 15–17, 22–24].

The mean AET also decreased significantly post-ARMS compared to pre-ARMS in 7 studies [11, 15, 17, 20–23]. Six of the following studies did not mention 24-h esophageal pH monitoring. Due to the discomfort and inconvenience for the patients during 24-h pH monitoring, it is difficult for the post-operative patients to follow up with the 24-h esophageal pH monitoring. For the researchers, improvement in the DeMeester score and AET is the main objective measurement after ARMS. For patients, improvements in symptoms are more important. The results of the tests can guide us in administering further treatment to alleviate the symptoms of the patient.

#### Flap valve score (Hill’s classification) (Fig. 7, [25]).

**Fig. 7:**
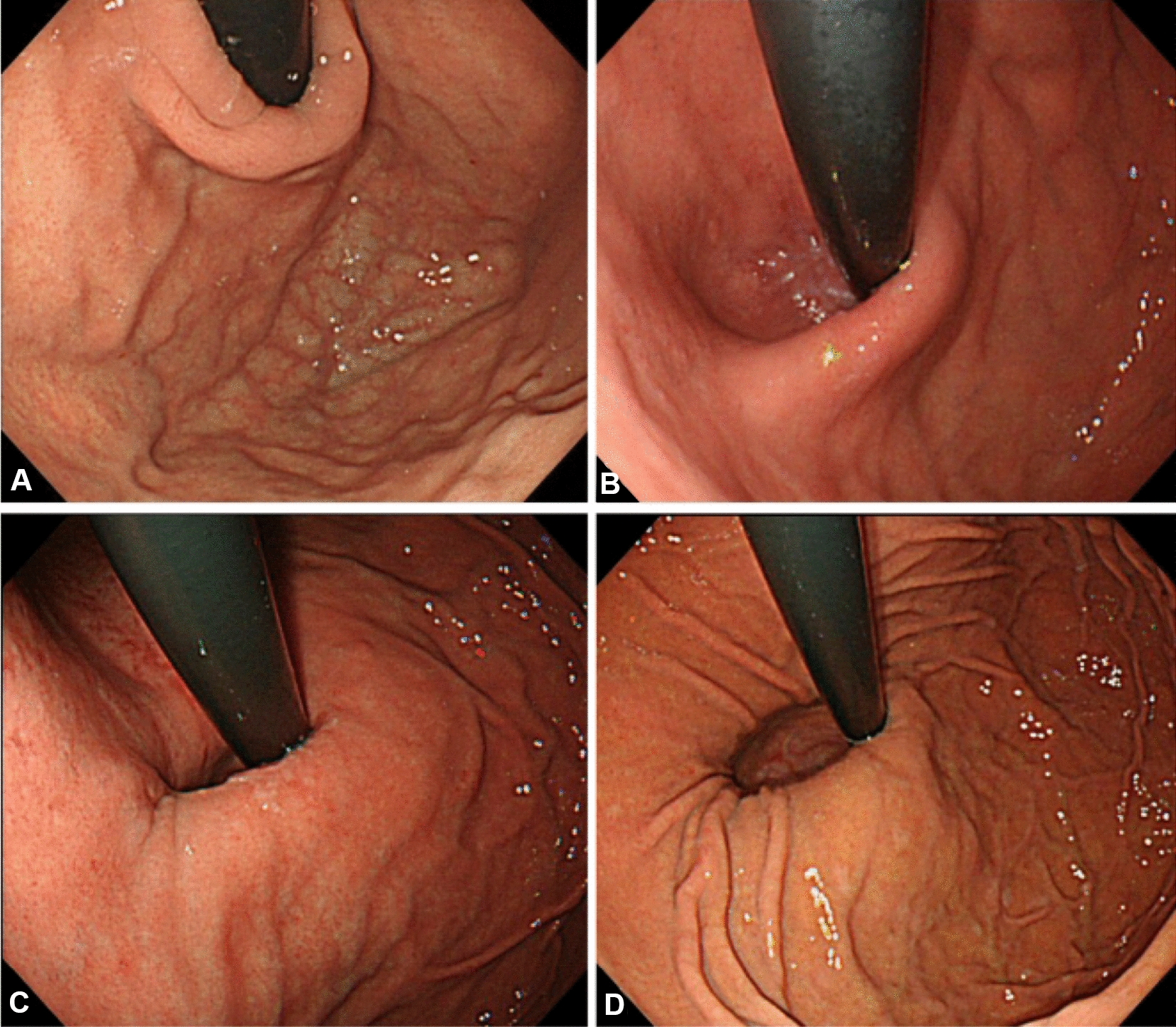
Flap valve score (Hill’s classification). **a** Grade I = a prominent fold of tissue along the lesser curvature next to the endoscope. **b** Grade II = the fold is less prominent with spontaneous openings and closings around the endoscope. **c**Grade III = the fold is not prominent, and the endoscope is not tightly gripped. **d**Grade IV = no fold is seen and the lumen of esophagus is open in retroflexion, hiatal hernia is always present

Hill´s classification is as follows: grade I = a prominent fold of tissue along the lesser curvature next to the endoscope; grade II = the fold is less prominent with spontaneous openings and closings around the endoscope; grade III = the fold is not prominent, and the endoscope is not tightly gripped, grade IV = no fold is seen, and the lumen of esophagus is open in retroflexion, hiatal hernia is always present. Seven studies evaluated the flap valve score. Almost all 7 studies [11, 14, 17, 20, 23, 26, 27] showed that the results of Hill’s classification were significantly reduced. The score of Hill’s classification is totally reduced to Grade I. Grading of the gastroesophageal flap valve is easy and offers useful information in the evaluation of patients undergoing endoscopy [28]. Morphologically, the lower flap valve score post-ARMS demonstrated the effectiveness of ARMS to some degree. It is one of the important criteria for postoperative evaluation.

#### The use of acid inhibitors

From the following studies in Table 1, we can also evaluate the efficacy of ARMS by the use of postoperative acid suppressants. Some patients can discontinue the use of acid inhibitors after ARMS, and some can reduce the dose of acid inhibitors by decreasing the dose or by intermittently using acid suppressants post-ARMS [16]. Only a small number of patients remain on the original dose post-ARMS.

## What will affect the efficacy of ARMS

### The quantity of mucosa to be resected

The quantity of mucosa to be resected to induce appropriate scar formation is an extreme issue in this operation. A tight gastroesophageal junction will require endoscopic esophageal dilation. A relatively loose gastroesophageal junction may have no anti-reflux effect.

#### Circumferential resection or crescentic resection (Fig. 8, [11, 20])

**Fig. 8:**
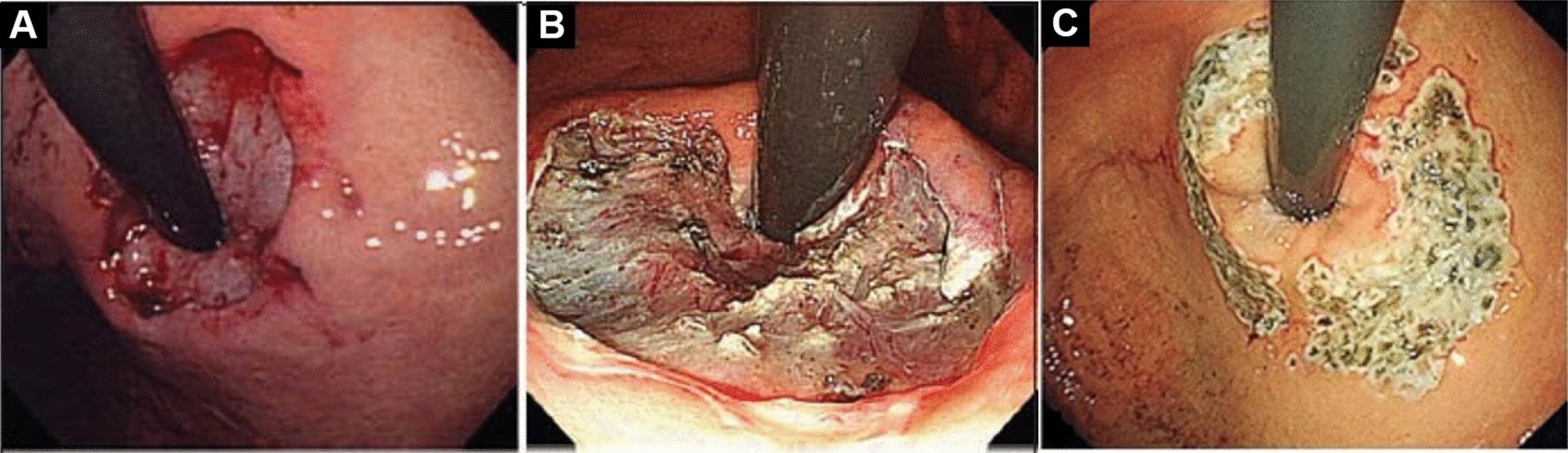
Three kinds of ARMS resection. **A** Circumferential resection.** B** Crescentic resection.** C** Butterfly-shaped resection

Inoue et al. [11] reported that the initial 2 cases of total circumferential resection required repeat balloon dilation to control post-ARMS stenosis despite symptom control. Subtotal dissection, also called crescentic dissection, not only managed the symptoms but also reduced the incidence of stenosis. It is suggested that there are several factors influencing the ideal range of mucosal reduction: the extent of laxity at the EGJ and esophageal contractile function. For example, half circumferential mucosal reduction should be used due to hypomotility of the esophageal body. Subsequently, many studies [16, 27] have followed crescentic resection. The study conducted by Yang et al. [28] showed there’s no significant difference between 180° ARMS and 270° ARMS regarding GERD-Q, quality of life, PPI use, gastroesophageal flap valve grade, presence of reflux esophagitis, acid exposure time (AET), distal contractile integral (DCI), and integrated relaxation pressure (IRP) and complication rate during the postoperative 6 months follow-up except for fewer complaints of newly dysphagia in 180° ARMS group. Gao et al. [29] reported that the GERD-Q, DeMeester scores and AET index improved at 6 months after operation in both the 2/3 and 3/4 circumference resection groups. However, the incidence of postoperative esophageal stenosis in the 3/4 circumferential mucosal resection group was higher than that in the 2/3 circumferential mucosal resection group.

#### Crescentic resection or butterfly-shaped resection (Fig. 8, [11, 20])

Sumi et al. [22] reported that patients who underwent butterfly-shaped resection had a lower risk of dysphagia than those who underwent crescentic resection. Only one of 21 patients who underwent ARMS with the butterfly method required balloon dilation, while 12 of 81 patients had stenosis after crescentic resection. Haruhiro et al. [20] reported leaving two contralateral areas of normal cardia mucosa with approximately one scope diameter to avoid stenosis when anti-reflux mucosal ablation (ARMA) was planned around the cardia in a butterfly shape. More high-quality studies are needed to further prove this hypothesis.

#### ARMS centered at the lesser curve or at the greater curve

ARMS centered at the lesser curve of the esophagogastric junctional (EGJ) mucosa has a lower incidence of requiring an additional anti-reflux operation [27]. Consequently, the mucosal flap valve at the greater curve was preserved [11]. More ARMS were centered at the lesser curve than at the greater curve. However, there is also a study involving mucosal resection at the greater curvature [14]. More studies are required to confirm this problem.

#### The length of mucosa to be resected

The length of ARMS may influence the outcomes of procedures. Inoue et al. [11] recommended that mucosal reduction was carried out in 1 cm esophageal site and 2 cm gastric side. They referred that the mucosal resection on the gastric side may contribute more to antireflux, and the overall length could also vary. So far, the studies on the influence of the length of mucosa to be resected on the efficacy of ARMS are rare. More high-quality studies are needed to provide us with further valuable information.

### Complications of ARMS

The complications of ARMS include immediate or delayed complications. Bleeding, perforation and infection are immediate complications. Of these, bleeding is more common. The most common delayed complication is esophageal stricture, in which the clinical manifestation is dysphagia. A systematic review and meta-analysis reported an 11.4% risk of dysphagia and a 5% risk of bleeding [13]. Mucosal resection, which involves more than three-quarters of the circumference of the squamous esophageal mucosa, might increase the risk of esophageal stricture [30]. According to Inoue, less squamous mucosa involvement in the resected area may decrease the risk of stricture. In addition, the rate of stenosis has significantly decreased by adopting butterfly-shaped resection, leaving the mucosa on both sides of the lesser and greater curves unresected rather than performing circumferential resection [31]. We should be able to individualize the length and quantity of resection to reduce the incidence of stenosis. Hedberg et al. [18] noted that the effect of gastric acid on scarification post-ARMS is another factor influencing the remodeling of the EGJ. This may result in a high incidence of stenosis when PPIs are discontinued immediately after the operation. It is suggested to keep taking PPIs for more than 2 weeks after the procedure to reduce inflammation and scarring and thereby the incidence of dysphagia. Most patients who have dysphagia or esophageal strictures post-ARMS may receive endoscopic balloon dilation to relieve symptoms. With the improvement of ARMS and well-experienced skills of endoscopists, the incidence of complications will decrease.

### The limitations and prospects of ARMS

Up to now, the population of studies on ARMS is relatively limited. Most studies [11, 18–20, 26, 27] excluded the patients who had a hiatal hernia longer than 2 cm (or Hill score > 3 or 4), while those are more likely to suffer from refractory esophagitis. Just a recently study [29] included the PPI-refractory GERD patients with a 3–5 cm hernia sac. As for the definition of refractory reflux esophagitis, there is no universal definition. Patients with refractory reflux included in the study were included according to different national standards. In addition, several studies [14, 18, 32] had no 24 h esophageal pH monitoring results neither pre-operation nor post-operation. Without pH-impedance recordings before the procedure, it was difficult to distinguish r-GERD patients from functional heartburn ones those did not need operation. For the same, there is no objective evidence for postoperative results without 24 h esophageal pH monitoring.

Currently, the number of studies on ARMS is relatively small. We need more large-sample research to evaluate the safety and efficacy of this procedure. Moreover, almost all the follow-up periods of the current studies range from several months to two years. The follow-up time needs to be prolonged to validate the long-term efficacy. More future prospective studies and comparisons to other treatments are needed.

ARMS seems to be an effective and well-tolerated endoscopic treatment strategy for refractory GERD. Due to its less invasive technique, it can fill the gap between acid inhibitors and laparoscopic fundoplication for the treatment of refractory GERD.

## Data Availability

Data sharing is not applicable to this article as no data sets were generated or analyzed during the current study.

## Abbreviations

GERD: Gastroesophageal reflux disease
ARMS: Anti-reflux mucosectomy
MSA: Magnetic sphincter augmentation
TIF: Traditional incisionless fundoplication
AET: Acid exposure time
EGJ: Esophagogastric junctional
GERD-HRQL: GERD-Health Related Quality of Life
GERD-Q: GERD-Questionnaire
Mon: Month
SD: Standard deviation
RAP: Resection and plication
PECC: Peroral endoscopic cardinal constriction
ARMS-C: Anti-reflux mucosectomy using cap-assisted endoscopic mucosal resection (EMR-C)
ARAT: Anti-reflux ablation therapy
ARMA: Anti-reflux mucosal ablation
ARMS-b: Anti-reflux mucosectomy band
